# Using Multi-Dimensional Dynamic Time Warping to Identify Time-Varying Lead-Lag Relationships

**DOI:** 10.3390/s22186884

**Published:** 2022-09-12

**Authors:** Johannes Stübinger, Dominik Walter

**Affiliations:** Department of Statistics and Econometrics, University of Erlangen-Nürnberg, Lange Gasse 20, 90403 Nuremberg, Germany

**Keywords:** time-varying lead–lag effect, dynamic time warping, data science, big data processing, multi-dimensional, thermal optimal path, simulation study, econometric modeling

## Abstract

This paper develops a multi-dimensional Dynamic Time Warping (DTW) algorithm to identify varying lead-lag relationships between two different time series. Specifically, this manuscript contributes to the literature by improving upon the use towards lead-lag estimation. Our two-step procedure computes the multi-dimensional DTW alignment with the aid of shapeDTW and then utilises the output to extract the estimated time-varying lead-lag relationship between the original time series. Next, our extensive simulation study analyses the performance of the algorithm compared to the state-of-the-art methods Thermal Optimal Path (TOP), Symmetric Thermal Optimal Path (TOPS), Rolling Cross-Correlation (RCC), Dynamic Time Warping (DTW), and Derivative Dynamic Time Warping (DDTW). We observe a strong outperformance of the algorithm regarding efficiency, robustness, and feasibility.

## 1. Introduction

**Figure 1:**
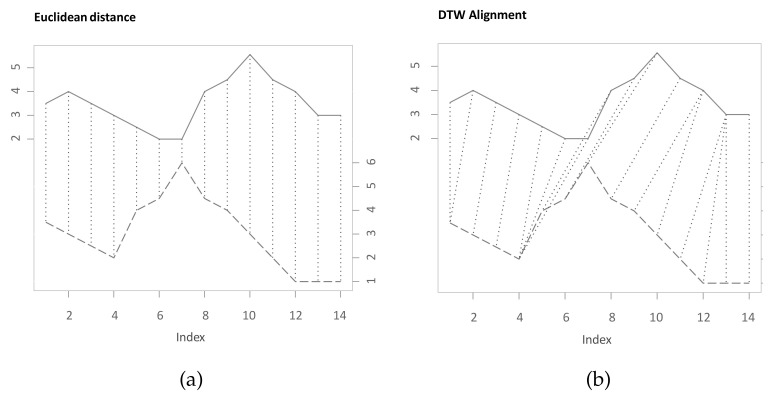
(**a**) Euclidean distance of the realizations of the two time series *X* and *Y*. (**b**) Dynamic Time Warping (DTW) alignment of the two time series *X* and *Y*.

For identifying similarities between two variables, it is often important to take their temporal developments into account. While analogies between time series might be contemporaneously, there are several cases where similar phases occur lagged or with different durations. To quantify similarities between time series, one common measure is the Euclidean distance. While it can be relevant to measure contemporaneous similarities in time series, the distance is not well suited in case of differences in the time dimension [1]. Dynamic Time Warping (DTW) is an algorithm that efficiently determines the nonlinear alignment of two time series that is optimal with respect to a set of criteria [2,3,4,5]. Thereby, the algorithm is not distorted through differences in the time domain, such as leads or lags. Figure 1 shows different alignments for two time series (X,Y) that both share common values and progressions, which, however, do not occur simultaneously. In Figure 1a, the presented alignment through the Euclidean distance is non-intuitive, and the time-shifted similarity between the time series is therefore underappreciated. Figure 1b depicts the capability of DTW to match and compare similar shapes and to thus quantify the similarity of these two time series more realistically. The comparison between Euclidean distance and DTW is also highlighted by Keogh and Pazzani [1]. Details about the theoretical concept, functionality, and application areas are described in Section 2.

This manuscript utilises a multi-dimensional DTW algorithm to identify the temporal divergence between two different time series. It aims at both finding similarities between time series and to computing an estimate of the potentially time-varying difference in timing for similar movements. This latter estimate is then referred to as lead-lag relationship as also done amongst others in Meng et al. [6]. The contributions of this manuscript to the literature are highlighted in detail in Section 2.4 after the review of relevant literature. To set the scene, this manuscript contributes by (a) improving upon the use towards lead-lag estimation of a recently introduced multi-dimensional DTW algorithm and (b) underlining the performance of the algorithm in comparison to other current methods in an extensive simulation study. Importantly, this manuscript does not try to answer the question of causality. The potential usefulness in further research is diverse. In case of stable or other predictable lead-lag relationships, where two variables have similar, yet temporally shifted, developments the leading variable might prove useful in forecasting the one that is lagging behind [7]. Moreover, strong lead-lag relationships between two time series can inspire further research on this potential relation. Finally, as a positive side effect, the assessment criterion in the simulation study might also be utilised to measure the time series alignment accuracy of different (DTW) algorithms.

The remainder of this manuscript is thereby structured as follows: Section 2 introduces the classical DTW algorithm, gives a review of relevant literature, and points the contribution of this manuscript out. Then, Section 3 presents the main methodology developed in this manuscript, followed by an introduction of alternative methods in Section 4. One major focus of this manuscript then is on the simulation study and comparison in Section 5, where the methods from Section 3 and Section 4 are evaluated on synthetic data. Moreover, Section 5 contains a real-world application on the business cycles of the US and Germany. Finally, Section 6 concludes and shows future research directions.

## 2. Literature Review of DTW

**Figure 2:**
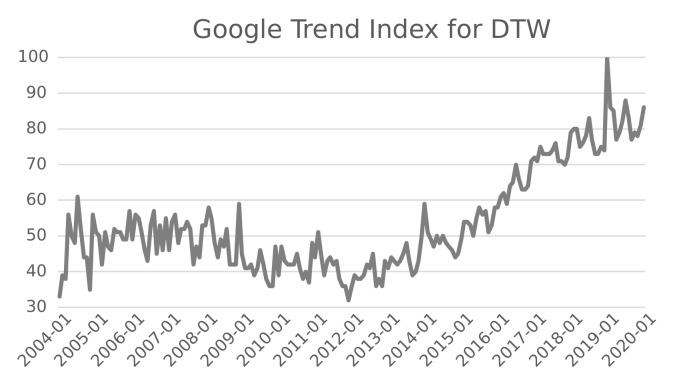
Google Trend Index for DTW from 2004 to 2020.

Dynamic Time Warping is becoming more and more popular. This statement is confirmed by Google Trends, which is a website that analyzes the popularity of top search queries in Google Search across various regions and languages. Figure 2 represents the Google Trend Index for DTW during the time span from 2004 to 2020. We observe a fluctuating interest until 2010 and a strong growth of attention during the last decade.

### 2.1. Concept of Classical DTW

The purpose of this section is to describe the general functioning of classical DTW. The notation and explanation of the classical DTW algorithm in this manuscript predominantly follow the one in Müller [2]. Additionally, the works of Senin [8], Giorgino [9] are also considered. DTW calculates the optimal mapping between two time series $X:=(x_{1},x_{2},\ldots ,x_{N})$ with N∈N and $Y:=(y_{1},y_{2},\ldots ,y_{M})$ with M∈N. DTW itself is based on a calculated local cost matrix that contains a positive measure of dissimilarity between all pairwise elements of *X* and *Y*. The dissimilarity value is often referred to as local cost and typical measures include Manhattan or Euclidean distance [2,8,9]:

(1) $$\begin{array}{c} C_{local}\in R^{N\cdot M}\;\mathrm{with}\;C_{local}\left(n,m\right):=c\left(x_{n},y_{m}\right),\\ \mathrm{Manhattan}\mathrm{distance}:\;c\left(x_{n},y_{m}\right)=\left|x_{n}-y_{m}\right|. \end{array}$$

**Figure 3:**
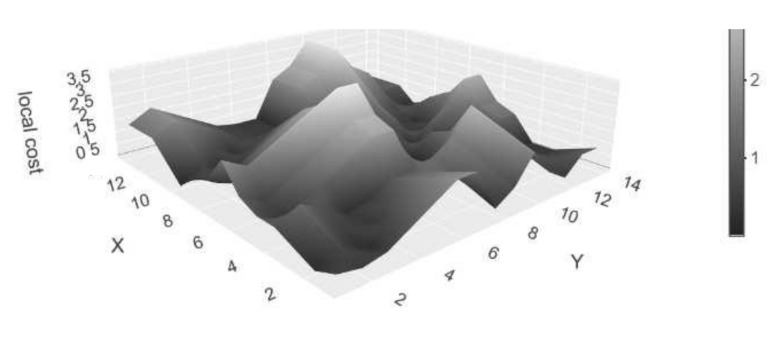
Local cost matrix of the time series *X* and *Y*.

Figure 3 shows the local cost matrix as a three-dimensional plot for the time series presented in Figure 1. For univariate time series, the Manhattan and Euclidean distance at each index pair give the same output. The distinction is only relevant in the multi-dimensional case [4].

Based upon the local cost matrix, the classical DTW algorithm searches for the best nonlinear alignment between both time series under three conditions. Each potential mapping that fulfils the three restrictions is referred to as warping path. A warping path $p=(p_{1},p_{2},\ldots ,p_{L})$ matches the indices of both series [2,8]:

(2) $$p_{l}=\left(n_{l},m_{l}\right)\in \left[1:N\right]\;\cdot \;\left[1:M\right]\;\mathrm{with}\;l\in \left[1:L\right].$$

The three conditions any warping path needs to fulfil are referred to as boundary, monotonicity and step size condition and defined as follows in [2]:

(a) **Boundary condition:**$p_{1}=\left(1,1\right)$ and $p_{L}=\left(N,M\right)$.
(b) **Monotonicity condition:**$n_{1}\leq n_{2}\leq \ldots \leq n_{L}$ and $m_{1}\leq m_{2}\leq \ldots \leq m_{L}$.
(c) **Step size condition:**$p_{l+1}-p_{l}\in \left\{\left(1,0\right),\left(0,1\right),\left(1,1\right)\right\}$ with l∈[1:L−1].

Following [2,8], the boundary condition enforces that both time series are mapped onto each other in their entirety in that both first elements and both last elements are matched, respectively. Later in the application on lead-lag relationships, the boundary condition is partly relaxed. As apparent to the interested reader, the step size condition already necessitates the monotonicity condition. Nevertheless, the monotonicity condition should be known as the basis for sensible step size conditions. This condition sustains the chronological order of the indices. The presented step size condition in combination with the boundary condition then intensifies the monotonicity condition by enforcing each index to be matched at least once and each index combination to be unique. Several alternative step size conditions that allow for skipping indices or restricting the number of repetitions of one single index are presented in further research and a short review thereof can be found in Giorgino [9]. However, for the remainder of this manuscript, the classical step size conditions are utilised [2,8].

According to Müller [2], the total cost of a warping path $c_{p}\left(X,Y\right)$ that aligns the two series *X* and *Y* is the sum of local costs along the warping path and can be written as:

(3) $$c_{p}\left(X,Y\right):=\sum_{l=1}^{L}c\left(x_{n_{l}},y_{m_{l}}\right).$$

The one warping path with the least total cost is then termed an optimal warping path. Moreover, this amount of total cost is then referred to as DTW distance [2]. Thereby, the DTW distance is not a distance metric as it does not necessarily fulfil the triangle inequality and thus is rather a measure of distance [2,3]. However, as Ref. Mueen and Keogh [3] notes, the non-fulfilment is a very unlikely case. Following [10], computational complexity is a major challenge in handling time series. The DTW method can be computed in an efficient manner through dynamic programming in O(N·M). In order to understand the procedure, ref. [2] defines prefix sequences of the two time series *X* and *Y* as $X\left(1:n\right):=\left(x_{1},x_{2},\ldots ,x_{n}\right)$ and $Y\left(1:m\right):=\left(y_{1},y_{2},\ldots ,y_{m}\right)$ with n∈[1:N] and m∈[1:M]. The accumulated cost matrix *D* contains the DTW distances between the prefix sequences for all possible combinations of *n* and *m* and thus exhibits the same dimensions as the local cost matrix. The accumulated cost matrix then logically contains the DTW distance between the entire time series *X* and *Y* in the cell, where the last row meets the last column, as then the respective prefix sequences correspond to the original time series. To efficiently compute the accumulated cost matrix from the local cost matrix, the following dynamic programming recursion is applied [2,8]:

(a) **First column:**$D\left(n,1\right)=\sum_{k=1}^{n}c\left(x_{k},y_{1}\right)$ with n∈[1:N].
(b) **First row:**$D\left(1,m\right)=\sum_{k=1}^{m}c\left(x_{1},y_{k}\right)$ with m∈[1:M].
(c) **Remainder:**$D\left(n,m\right)=min\left\{D\left(n-1,m-1\right),D\left(n-1,m\right),D\left(n,m-1\right)\right\}+c\left(x_{n},y_{m}\right)$.

**Figure 4:**
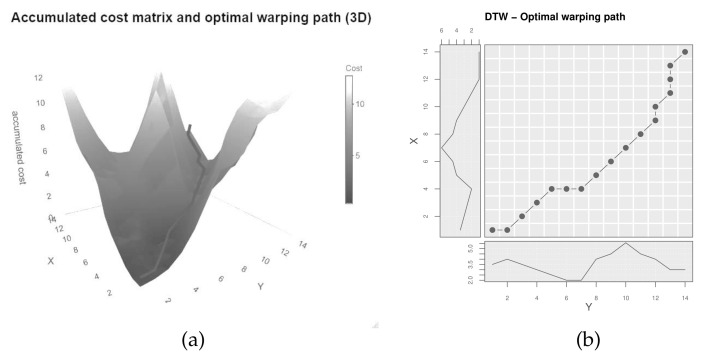
(**a**) accumulated cost matrix of the two time series *X* and *Y*; (**b**) optimal warping path of the two time series *X* and *Y*.

The accumulated cost matrix therefore builds up through adding the current local cost to the warping path with the minimal cost up to the current cell. As Giorgino [9] mentions, this recursion prefers one diagonal over the combination of one top and one right. By memorizing the steps taken, the optimal warping path can be retraced starting at (N,M) [2,8]. Figure 4a shows the accumulated cost matrix in 3D with the optimal warping path (dark line), Figure 4b depicts the optimal warping path projected onto the plane, aligning the indices of the original time series.

### 2.2. Relevant Adaptions to DTW

Since the introduction of classical DTW, several adaptions to the algorithm have been published. Following the review in Müller [2], the adaptions focus to a large degree on improving run-time while trading off the guarantee of a globally optimal warping path (global constraints, approximations or Multiscale DTW) or to improve the alignment appropriateness in case of a priori knowledge about sensible alignments (further step size conditions or local weightings).

The aim of this manuscript is to utilise the alignment path for inferring a lag structure of similar temporal developments. Since the algorithm is applied to few time series and no a priori knowledge is assumed, the above named adaptions would rather be of little use. This section therefore reviews literature on improving the alignment quality without the need for prior assumptions upon the route of warping paths.

One influential paper by Keogh and Pazzani [11] introduces Derivative DTW (DDTW) as a modification of DTW with the aim of improving alignment accuracy. Following Keogh and Pazzani [11], classical DTW struggles in aligning phases, where two time series exhibit differences in values for similarly shaped progression. DTW then compensates by warping the two time series and matching a single index in one time series with several from the other. This phenomenon is referred to in the literature as singularities. Furthermore, DTW might map indices with a small difference in absolute values, but with distinct underlying time trends. Therefore, the authors suggest performing DTW on the derivatives of the original time series, where Keogh and Pazzani [11] utilises the following equation to estimate the derivatives:

(4) $$D_{X}\left[x_{n}\right]=\frac{\left(x_{n}-x_{n-1}\right)+\left(\left(x_{n+1}-x_{n-1}\right)/2\right)}{2}\;\mathrm{with}\;n\in \left[2:N-1\right].$$

As described in Keogh and Pazzani [11], once the original time series are replaced respectively by their estimated derivatives, the remaining steps are equivalent to classical DTW. In contrast to classical DTW, DDTW does not consider the values of the time series, but rather local shape features. As Keogh and Pazzani [11] show for simulated data, DDTW can improve the alignment quality compared to DTW. Different computations of the derivatives can be found in [12,13].

The following adaptions upon increasing the alignment accuracy depend on an understanding of multi-dimensional DTW, sometimes also termed multivariate DTW as in Moser and Schramm [14], which stands for the use of DTW on multi-dimensional time series $X:=(x_{1},x_{2},\ldots ,x_{N})$ with $x_{n}\in R^{d}$ and $Y:=(y_{1},y_{2},\ldots ,y_{M})$ with $y_{m}\in R^{d}$ and d∈N,d≥2 [4,15]. While DTW is applied to multi-dimensional sequences starting with its introduction and ten Holt et al. [16] refer to their presented approach as multi-dimensional DTW already in 2007, the naming convention for potential variants of multi-dimensional DTW is introduced by Shokoohi-Yekta et al. [15]. Following Shokoohi-Yekta et al. [15], independent multi-dimensional DTW calculates the optimal warping path and DTW distance for X and Y separately for each of the *d* dimensions. Finally, the independent multi-dimensional DTW distance then is the sum over these *d* DTW distances [15].

Since this manuscript is concerned with the identification of lead-lag relationships through the optimal warping path, the second variant, dependent multi-dimensional DTW as described in Shokoohi-Yekta et al. [15] is more appropriate. This variant aggregates the information contained within the *d* dimensions and constructs one single local cost matrix and thus one single optimal warping path. The local cost matrix is generated by calculating the pairwise distances between all indices of the multi-dimensional time series [15]. The approach presented in ten Holt et al. [16] termed multi-dimensional DTW corresponds to dependent multi-dimensional DTW.

While DDTW addresses the emergence of singularities occurring through classical DTW, Xie and Wiltgen [13] show in an exemplary way that DDTW might fail to align common features between time series. Thus, Xie and Wiltgen [13] suggest extending the information contained in global features of the series such as the values in DTW with local shape characteristics as depicted by DDTW. The idea of combining DTW and DDTW can also be found in ten Holt et al. [16], Kulbacki and Bak [17], Benedikt et al. [18], Górecki and Łuczak [19], Łuczak [20]. The approaches differ firstly in whether the contribution of DTW and DDTW towards the outcome is respectively weighted and, secondly, in the way the global feature and local feature is calculated. Thirdly, and finally, while Górecki and Łuczak [19], Łuczak [20] separately perform DTW on the original series and the differentiated series as in independent multi-dimensional DTW, the other approaches aggregate both global and local features in one local cost matrix as in dependent multi-dimensional DTW. Through concatenating the sequences of local and global features as separate dimensions, the resulting sequence can be used as input to dependent multi-dimensional DTW. This follows the idea of Zhao and Itti [4], ten Holt et al. [16].

A further enhancement on the classification accuracy and alignment quality is presented in Zhao and Itti [4]. The authors present their algorithm shapeDTW, which represents a flexible framework for aggregating global and local information in the local cost matrix. The framework includes DTW, DDTW and a combination of DTW and DDTW as special cases and extends these ideas by explicitly including information on neighbouring points in the pairwise distance calculation of the local cost matrix. shapeDTW is thereby based on two key steps. In the first step, the original time series is transformed to vectors representing not only the original point at each index, but also neighbouring points. The two univariate time series are thus converted into multi-dimensional sequences describing the original series. In the second step, the two multi-dimensional sequences are aligned in the spirit of dependent multi-dimensional DTW [4]. Approaches considering neighbourhood information in the construction of the local cost matrix can also be found in Skutkova et al. [21], Zhang et al. [22], Folgado et al. [23]. Generally, DDTW by Keogh and Pazzani [11] and the approach by Xie and Wiltgen [13] also indirectly use information of neighbouring points in the way the distance is calculated, yet from only one neighbouring point in each direction. shapeDTW in contrast explicitly uses a free determinable number of neighbouring points both with their value and optionally with their local shape [4].

To calculate the descriptors following Zhao and Itti [4], subsequences of length *l* (l∈N and lmod2=1 to ensure symmetry) centred at each index respectively are extracted. For the input series $X:=(x_{1},x_{2},\ldots ,x_{N})$ with N∈N, the descriptor at index *i* is calculated in two steps. Firstly, the subsequence $X\left(\left(i-\frac{l-1}{2}\right):\left(i+\frac{l-1}{2}\right)\right)=\left(x_{i-\frac{l-1}{2}},\ldots ,x_{i},\ldots ,x_{i+\frac{l-1}{2}}\right)$ is extracted. The descriptor for index *i* is then either identical to the subsequence or the subsequence is further processed. For the latter, Zhao and Itti [4] present five different processing possibilities that further transform the subsequences. One of these is the estimation of the derivative as in DDTW. Moreover, Zhao and Itti [4] suggest to potentially concatenate more than one representation of the subsequence to combine their informative values. The other presented transformations additionally focus on reducing the dimensions of the descriptor. Importantly, if the subsequence length *l* is set equal to 1, then a descriptor equal to the subsequence itself reduces to classical DTW, the subsequence processed as derivatives reduces to DDTW and a concatenation of both reduce to be conceptually similar to Xie and Wiltgen [13]. Therefore, the difference of shapeDTW comes into effect for values of l>1. Zhao and Itti [4] show that shapeDTW improves the alignment quality for simulated time series with known underlying alignments in comparison to DTW and DDTW and outperforms DTW as a Nearest-Neighbour classifier on 64 from 84 UCR classification datasets published by Chen et al. [24]. Following Zhao and Itti [4], shapeDTW has a preceding step of extracting the descriptors, which approximately takes time O(l·L), where *l*  is the length of the subsequence and *L*  the one of the time series. Along with the second step of computing the DTW alignment, shapeDTW runs in $O(l\cdot L^{2})$ for time series of equal-length.

### 2.3. Applications of DTW

As noted by Gasser and Wang [25], Keogh and Ratanamahatana [26], DTW has its main origins in speech recognition. Common references are Rabiner et al. [27], Sakoe and Chiba [28], Myers et al. [29] that cover the identification of isolated words through comparing new recordings against references with the help of DTW. The importance of DTW’s invariance to different timings becomes clear, when envisioning the various speaking rates and emphasis while pronouncing the exact same word in different situations. As Ratanamahatana and Keogh [30] note, the applications of DTW cover classification, clustering and identification of anomalies. In its application to classification of time series, DTW combined with Nearest Neighbour has long been in a leading role, with classification algorithms failing to significantly beat DTW [31,32].

To gain further insight upon DTW’s use in practice, recent areas of application are presented exemplary in this paragraph. Ahmed et al. [33] use DTW to recognise hand movements of sign language from video footage. Another application to gesture recognition can be found in Calin [34], where movements of Xbox Kinect devices are classified. DTW is also applied in ensuring signature authenticity, as in Al-Hmouz et al. [35]. Further applications include trading strategies based on DTW [36] and automotive uses, where Moser and Schramm [14] review published applications. Jeong et al. [37] apply DTW to verify the effectiveness of DTW for time series classification and clustering. Specifically, the data set includes real-life time series, synthetic time series, and generic time series that come from different application domains and are obtained from UCR Time Series Data Mining Archive, e.g., fish and beef.

### 2.4. Lead-Lag Relationships with DTW

Before introducing literature upon lead-lag relationships based on DTW, this paragraph briefly introduces this research strand. Trying to understand relationships between different variables concerns many parts of research. Focusing on relationships with temporal divergences in the sense that one variable lags another has been addressed according to Sornette and Zhou [38], Yue et al. [39] with methods such as cross-correlation, Granger causality or time-distance as introduced in Granger and Jeon [40]. However, as Sornette and Zhou [38] elaborate, cross-correlation misses nonlinearities in the relationship and both cross-correlations and Granger causality require a lot of data to draw conclusions. Additionally, Granger causality is often based on parametric models. The following presented research line is non-parametric and aims at detecting time-varying lead-lag relationships accurately. Importantly, none of these methods, including the one presented in this manuscript, tries to answer the question of underlying causality between variables. They rather aim at identifying similarities and lead-lag relationships between different time series. The term lead-lag relationship refers to differences in timing of similar progressions in two different time series [6,38].

The idea of applying DTW for determining lead-lag relationships is already presented in Varfis et al. [41]. The authors present their novel methodology and apply it to both synthetic and real data. Varfis et al. [41] moreover demonstrate how to translate the optimal warping path into an estimator of the time difference between the matched indices. This transformation is also central to the main methodology detailed in Section 3. The underlying DTW algorithm in Varfis et al. [41] corresponds to classical DTW with an inclusion of a warping window limiting the differences between matched indices. While this can reduce excessive warping and run-time, warping windows restrict potential lead-lag values that can be found and thus might prevent the algorithm from identifying the globally optimal matching between the two time series. Moreover, as the algorithm is applied to two input series, the run-time saved is negligible and does not overcompensate the potential loss of a global optimum [41].

Four years later, Sornette and Zhou [38] introduce the optimal thermal causal path method, which exhibits methodological similarities to the DTW algorithm. While the authors as well as Gupta and Chatterjee [42] mention an underlying commonness, the method is generally not referred to as a DTW algorithm, since the idea of averaging over several warping paths is distinctly different to DTW. Therefore, the algorithm from Sornette and Zhou [38] and resultant improvements are described in Section 4 and are additionally later used as comparison for the presented main methodology.

Stübinger [43] uses a comparable approach to DTW for applying it to a statistical arbitrage method. The approach focuses on the identification of stable lead-lag relationships between stock pairs. For the pairs with the steadiest relations in the in-sample period, the leading stock is then used as a trade signal for the lagging stock out-of-sample. The market and currency risks are offset through an opposite investment into the S&P 500 [43].

Further research on lead-lag relationships applying DTW include Taylor et al. [44], who analyse leads from one car that drives behind another. Claure et al. [45] examine the temporal difference in the amount of water between connected rivers and Woo et al. [46] in a similar spirit investigate temporal progressions of tracers within water networks. Both of the latter apply slightly modified versions of DDTW as algorithms. In another very specific application, Gao et al. [47] use a combination of DTW and DDTW in examining the spreading of topics among different online platforms. Li et al. [7] use a comparable approach in predicting how popular certain topics get in social media. Finally, Franses and Wiemann [48] show, for US business cycle data, how the DTW approach by Xie and Wiltgen [13] can be applied to visualise whether one state leads or lags another. As can be seen, the application of DTW upon lead-lag relationships is a topic of current research.

While the research presented above based their analysis of the lag structure upon a local cost matrix that is calculated as in classical DTW, DDTW or a modified combination thereof, there has only been little further research applying approaches similar to the idea of shapeDTW and the respective papers are very recent. To the best of our knowledge only Gupta and Chatterjee [42], Ito and Sakemoto [49], Gupta and Chatterjee [50] detail similar approaches. Ito and Sakemoto [49] calculate the local cost matrix from rolling windows over non-synchronous financial time series. The approach, however, suffers from several drawbacks. Firstly, indications on choosing the optimal window size are given for stable lead-lag structures and not time-varying lag structures. Secondly, no relaxation of the boundary condition from DTW is proposed to reduce the bias at the begin and end. Thirdly, the time scale of the outputted lag structure is not transformed to the inputted time scale, which renders the interpretation more difficult. Fourthly, the algorithm is based on an approximation to DTW named FastDTW, which may not compute the globally optimal alignment and no test of significance of the outputted lead-lag structure is deployed [49].

Gupta and Chatterjee [42,50] also propose a detection for discovering lead-lag relationships over rolling subsequences in similar spirit to shapeDTW. However, it uses a correlation-based distance measure instead of Euclidean or Manhattan distance, which therefore cannot be reduced to classical DTW and does not work for small subsequence lengths. Therefore, the two papers set the subsequence length to values of at least 25, which might miss higher-frequency changes in the lead-lag relationship.

Finally, it is to be noted that none of these papers cites the concept of shapeDTW presented already in 2018 by Zhao and Itti [4]. This manuscript firstly contributes to the literature by providing a more holistic picture of the use of neighbourhood information in the spirit of Zhao and Itti [4] within the determination of lead-lag relationships. Even though the general methodology is similar for this manuscript, for Gupta and Chatterjee [42,50] and for Ito and Sakemoto [49], this manuscript thoroughly provides a testing of the single parameter (subsequence length) for time-varying lead-lag relationships, provides a more advanced method of relaxing the boundary constraint in comparison to the compared research, describes the conversion to the original time scale and uses the globally optimal warping path. Moreover, this manuscript is unique among the compared papers by following the capabilities of shapeDTW in concatenating values with local shape features in the spirit of Zhao and Itti [4], Xie and Wiltgen [13]. This manuscript then secondly contributes by performing a large-scale simulation study with an assessment criterion based on different strength of underlying relationships and an extensive comparison to similar methods. As the main methodology of this manuscript and the papers by Gupta and Chatterjee [42], Ito and Sakemoto [49], Gupta and Chatterjee [50] share the same core, the methods by these authors are not considered in the comparison. This manuscript rather connects to this research strand, and subsequently improves and extends it in the many respects mentioned. Moreover, this manuscript then enhances the simulation study by various robustness checks towards scaling the relationship transmission, autoregressive noise and cyclical lag structures. One remaining issue for a future publications, which is already mentioned in Varfis et al. [41], is to find and apply appropriate significance tests, as DTW outputs an optimal warping path and therefore lead-lag estimate for any two series, independent of any sensible connection. Even for series such as the stock price of Apple and rainfall in Hamburg, DTW would output a lead-lag relationship estimate.

The most prominent method to identify time-varying lead-lag relationships in recent research is probably the “optimal thermal causal path method” also referred to as “Thermal Optimal Path method” [6,38] and its revision named “Symmetric Thermal Optimal Path method” [6]. Several papers have used the method in economic and financial applications [51,52,53].

The TOP and TOPS method show conceptual similarities towards DTW. These two former are equally based on a local cost matrix, which is referred to as “distance matrix” or “energy landscape” by Meng et al. [6], Sornette and Zhou [38]. The authors calculate the local cost matrix as in the case of the classical DTW. They then introduce a parameter referred to as temperature *T*, which allows for extracting information of multiple warping paths for T>0. For a value of T=0, the TOP method corresponds to classical DTW in finding an optimal warping path between prefix series, yet not the entire series. This is explained in the lead-lag estimation at each time for the TOP method only depending on previous observations. Thus, the lead-lag estimates at T=0 rather likely do not fully correspond to the globally optimal warping path of classical DTW that considers the entire time series.

The then further explicit distinction from DTW occurs for values of T>0. Then, TOP and TOPS do not only take into account the optimal warping path but also warping paths with total costs above the minimum. As in the main methodology presented in Section 3, Meng et al. [6], Sornette and Zhou [38] define the deviations from the diagonal as measure of the leads or lags. They then calculate an average of the potential leads or lags at each line perpendicular on the diagonal, weighted by the likelihood that warping paths go through the respective cell. The probability is calculated by weighting all allowed warping paths with a factor of $e^{\frac{-\Delta E}{T}}$, where ΔE measures the accumulated costs above the minimal accumulated costs, and *T* depicts the temperature. The difference of the TOPS methodology compared to TOP is that both the accumulated costs of all potential warping paths from the beginning up to the respective cell as well as the accumulated costs from there up until the end contribute equally to the weighting, while for the TOP method only the accumulated costs from the start to a respective cell are considered [6,38].

The higher the value of *T*, the higher the contribution of warping paths with accumulated costs above the minimum up until each perpendicular. Meng et al. [6], Sornette and Zhou [38] suggest taking a value of *T* close to 2. Following Sornette and Zhou [38], the aim of introducing the parameter *T* is that, since the local cost matrix likely includes noise next to useful information on the lead-lag relationship, sampling the final lag structure over several warping paths can reduce the dependence on noise. Analogously to the weighted averaging of the lead-lag estimates, the TOP and TOPS method further calculates a normalized weighted average over the local cost matrix. This is the comparable idea to the normalized DTW distance. Through the normalization, it is ensured that the similarity measure is comparable over a time series of different lengths or different start- and endpoints [38].

## 3. Main Methodology (MM)

This chapter focuses on the methodology based on dependent multi-dimensional DTW to identify time-varying lead-lag relationships (see Section 3.1). Next, Section 3.2 presents the used software and Section 3.3 gives a detailed example.

### 3.1. Theoretical Concept

Our developed methodology can be split into two main steps. While the first main step (Section 3.1.1) is concerned with the computation of the multi-dimensional DTW alignment in the spirit of shapeDTW by Zhao and Itti [4], the second main step (Section 3.1.2) then utilises the output from the DTW alignment to extract the estimated potentially time-varying lead-lag relationship between the original time series. For the sake of clarity, the whole methodology is displayed in Algorithm 1.
**Algorithm 1** Main methodology to identify time-varying lead-lag relationships.** procedure**Main step 1: Compute multi-dimensional DTW alignment (Input:  two stationary univariate time series).  **Step 1** Z-normalize both time series.  **Step 2** Convert univariate time series to multi-dimensional descriptor series        as in shapeDTW [4].    **Step 2.1** Pad beginning and ending of time series by repeating the first and last           element $\left(\frac{l-1}{2}\right)$ times, respectively.    **Step 2.2** At each original index *i*, extract the subsequence of length *l* centred           at *i*.    **Step 2.3** Take each subsequence as vector for describing the respective index.    **Step 2.4 Optionally** concatenate subsequence with its first difference to          include shape characteristics invariant to the level of the values.  **Step 3** Z-normalize each dimension to allow for equal contribution as in       Shokoohi-Yekta et al. [15].  **Step 4** Calculate the local cost matrix using dependent multi-dimensional DTW.  **Step 5** Perform DTW on the local cost matrix, while **optionally** relaxing the        boundary condition.  **Output** DTW distance, optimal warping path, accumulated cost matrix,        local cost matrix, optimal start- and endpoint.**end procedure****procedure** Main step 2: Extract lead-lag relationship (Input: optimal warping path, optimal start- and endpoint).  **Assumption** Relationship of the form Y(j)=c+a·X(j−τ(j))+η,           $with\;\eta ∼N(0,\sigma_{\eta })$ in similar spirit to Sornette and Zhou [38].  **Step 1 If relaxed boundary condition,** transfer optimal warping path to       indices of original local cost matrix.  **Step 2** Subtract matched indices of the optimal warping path.  **Step 3** For each index of *Y*, take a value that comes last in the optimal warping path.   **Output** Identified lead-lag relationship.**end procedure**

#### 3.1.1. Main Step 1: Compute Multi-Dimensional DTW Alignment

The first step takes two stationary univariate time series as input, where stationary is meant in the weak sense. Thereby, the two time series can generally be of differing lengths. To make the input time series more comparable and invariant to potential differences in offset and variance, the time series are normalized. The importance of normalization for differences in the means between time series is elaborated in Keogh and Kasetty [54]. As Rakthanmanon et al. [55] state, Z-normalization is the most common normalization used in research with DTW. The aim of Z-normalization is to convert time series to a common scale by subtracting from each time series its respective mean and then dividing it by its standard deviation. As a result, both time series share a mean of 0 and a standard deviation of 1 [55].

In the second step, the normalized time series are converted to multi-dimensional descriptor sequences following the shapeDTW framework presented by Zhao and Itti [4]. The multi-dimensional time series share the same indices as their respective original time series. In order to extract subsequences at the beginning or the end of the univariate time series, these are padded by repeating the first and last element $\left(\frac{l-1}{2}\right)$ times, respectively. Then, subsequences of length *l* centred at the respective indices of the original time series are extracted. The length *l* is the parameter of the main methodology that will later be varied. The sequence of extracted vectors then forms the multi-dimensional descriptor series. Thus, each index is not only depicted through its value but also through the values of neighbouring points. This corresponds to the “Raw-Subsequence” version of the shapeDTW framework [4] (p. 174). The then computed local cost matrix exhibits relatively low values for index combinations where the extracted sequences at both indices are similar and relatively high values for dissimilar subsequences. The similarity is typically measured through the Manhattan or Euclidean distance between the vectors. This manuscript uses the Manhattan distance, such that the differences in each dimension contribute equally and the distance is not dominated by dimensions with larger differences. Optionally, as mentioned by Zhao and Itti [4], the extracted subsequences can be concatenated with further features that describe the extracted subsequence, such as localized measures of change as derivatives in Keogh and Pazzani [11] or the first difference as in Xie and Wiltgen [13]. Concerning different specifications of measurement of local change, Górecki and Łuczak [19] conclude that, in the application for classification, the outcomes only differ marginally. In order to be able to isolate the effects of neighbourhood information at each point from the effects of additionally including measures of change, the latter are only calculated based on the already extracted subsequences. As the measures are based on more than one point, their potential benefit might otherwise be explained through increased neighbourhood information. Therefore, this manuscript optionally concatenates the actual subsequence with the first difference of the subsequence in similar spirit to Xie and Wiltgen [13].

As a third step, each dimension is individually Z-normalized as suggested by Shokoohi-Yekta et al. [15]. In case the multi-dimensional sequence only consists of the extracted subsequences, this step is of minor importance, since each dimension—neglecting the starting and ending—consists of the already Z-normalized input time series shifted backwards or forwards in time. However, in case of the inclusion of the first difference, Z-normalizing allows for equal contribution of all dimensions. As noted by Zhao and Itti [4], each dimension or each set of related dimensions (values, first differences) can be weighted differently to control the contributions. However, this manuscript continues with equally weighting each dimension in order to keep the parameters of this method low and to avoid criticism of hyperparameter tuning.

Next, in the fourth step, the local cost matrix between the two multi-dimensional sequences is computed in the spirit of dependent multi-dimensional DTW [15]. For every possible index combination, the Manhattan distance between the respective vectors is calculated and stored in the local cost matrix.

In the final step, the local cost matrix is then used to calculate the DTW alignment between the two time series. There is, however, one drawback in utilising the standard DTW algorithm. Since the aim is to align indices with similar progressions and both time series do not necessarily start and end with contemporaneously similar phases, the imposed boundary condition forces the mapping of the two first indices and the two last indices. If for instance one of the time series is consistently leading the other one by five time periods, then for two time series with equal periodicity, the first five indices of the latter should not be matched as they would be related to the five indices of the former that occurred before the available time series start. Moreover, the undesirable matching of unrelated indices through the boundary condition increases the DTW distance disproportionately and most likely biases the warping path beyond the unrelated indices in case the transition to the “true” underlying lag structure is more costly for a while than continuing a different path [56]. Even though the framework in Sornette and Zhou [38] differs partly from DTW, the idea they present for relaxing the endpoint constraint is intuitive and can be seen as a compromise between the ideas of Silva et al. [56], Tormene et al. [57]. The authors allow the warping path to begin and end in a predefined set of cells in the first row or column and last row or column, respectively. They then calculate a normalized averaged energy for each combination of start- and endpoint and choose the start- and endpoint with the lowest normalized averaged energy. The endpoint relaxation applied in this manuscript then follows the idea of Sornette and Zhou [38] transferred to the DTW framework by considering the normalized DTW distance calculated in the spirit of Giorgino [9], Tormene et al. [57] instead of the normalized averaged energy. By relaxing the endpoint constraint in this manner, both time series may be truncated, the truncation is restricted, through normalization, the method does not bias shorter warping paths and the procedure is symmetric, if the set of start- and endpoints is the same for both series. In order to determine a suitable set of starting and ending points, it makes sense to choose a symmetric set, such that, for either series, the same amount can be truncated. Sornette and Zhou [38] describe the effects of increasing equal-sized sets of start- and endpoints on the run-time as quadratic. For DTW, this effect on the run-time can be reduced to nearly linear, as the local cost matrix only needs to be separately calculated for each startpoint. The normalized total costs for various endpoints can then be extracted after dividing the local cost matrix by the matrix of normalization factors as detailed in Tormene et al. [57]. Then, for each startpoint, the endpoint with minimal normalized total cost is memorized and finally the pair of start- and endpoint with the minimal values among those is considered as the optimal start- and endpoint.

#### 3.1.2. Main Step 2: Extract Lead-Lag Relationship

For extracting an estimate for the time-varying lead-lag relationship, the second main step requires the optimal warping path and the position of the optimal start- and endpoint. This manuscript follows the notation in Meng et al. [6], Sornette and Zhou [38] for the formulas on lead-lag relationships. The second main step assumes a relationship between the two time series of the form Y(j)=c+a·X(j−τ(j))+η with a>0, where τ(j) portrays the sought lag structure at each index of *Y* similarly to Meng et al. [6], Sornette and Zhou [38]. One assumes that *Y* at index *j* depends on *X* at index j−τ(j). For τ(j)>0(τ(j)<0), the methodology estimates that X(Y) is leading Y(X) by |τ(j)| time steps. τ(j)=0 points to contemporaneous movements. Thereby, τ(j) can be varying over time [6,38].

In the first step, the optimal warping path needs to be transferred to the original local cost matrix in case the boundary condition is relaxed. This is due to the fact that the relaxation of the boundary condition potentially truncates the original local cost matrix. Therefore, a warping path starting at (1,1) within this truncated local cost matrix refers to another index combinations within the original local cost matrix. If, for instance, the optimal startpoint is at (4,1), then the optimal warping path is relocated respective to this startpoint and not to (1,1). Thus, the optimal warping path then depicts the proposed matching between original indices of both time series before any truncation.

In the second step, the difference between the matched indices is calculated. This step is identical for nearly all literature using DTW for lead-lag relationships and thus amongst others found in Varfis et al. [41], Ito and Sakemoto [49]. A difference in indices suggests that one time series is leading the other, since the optimal warping path matches those indices that share similar progressions. This difference is a first estimate of the lead-lag structure because the differences depict the sought leads or lags. As the optimal warping path contains at least as many index pairs as the number of indices of the longer of the two time series and most probably more in case the longer series is warped, the lag structure needs to be transformed to the original time scale. Varfis et al. [41] already note the importance of this transformation. The methodology aims at identifying the appropriate τ(j) at each index of *Y* and if an index of *Y* is matched with multiple indices of *X*, the difference with the latest or rather highest index of *X* is taken. This is in similar spirit to Varfis et al. [41], where diagonal developments in the lag structure due to stretching of the template series are transformed to vertical. The idea of the explanation presented for this manuscript is that the optimal warping path, restricted through the step-size condition, faces prolonged transitions in case of jumps in the underlying lag structure. Thus, for each index of *Y*, the latest matched index of *X* allows for reducing prolongations in the transitions. Moreover, this reduced number of index combinations, and thus differences between them, matches the length of time series *Y*. While this step has benefits attached to it, it is this last step that makes the main methodology not fully symmetric. However, this caveat is accepted considering the presented advantage. Moreover, within the simulation study, an elegant solution upon achieving symmetry of this main methodology for equal-length time series is presented. Finally, the differences between the reduced index combinations output the final estimate of the lead-lag relationship.

### 3.2. Software

To implement the main methodology from Section 3 and the alternative methods described in Section 4, and to perform the simulation study in this chapter the statistical software R in version 4.0.5 is utilised [58] with RStudio from the US-American developer RStudio PBC in version 1.4.1106 as integrated development environment [59] on Windows 10 Home. Most of the coding is thereby self-implemented due to a lack of existing packages. The own implementation is almost exclusively done through self-written formulas with options (see Section 3). Based on the standard arima.sim function, one function allows for generating time series with inserted lead-lag relationship for given parameters with options for including transitions, scaling the transmission, including cyclical lags and different noise specifications. The second self-implemented main function takes the original time series and generates the multi-dimensional sequences. Finally, the local cost matrix is computed with the help of **proxy** by Meyer and Buchta [60], and the DTW alignment based on the local cost matrix is then undertaken through the package **dtw** (see Giorgino [9]). Different options for relaxing the boundary condition are also self-implemented. Moreover, **dtw** by Giorgino [9] and **plotly** by Sievert [61] are used to create graphics, **zoo** by Zeileis and Grothendieck [62] to calculate functions over rolling windows and deal with missing data and **tseries** by Trapletti and Hornik [63] for the stationarity tests.

### 3.3. Example

To gain an overview over the methods, an example application is performed, before the next sections then present the results of the in-depth simulation study. In an effort to generate synthetic time series with underlying time-varying lead-lag relationship, this manuscript follows the approach and notation presented in Meng et al. [6], Sornette and Zhou [38]. Therefore, the first time series X(i) is simulated as an AR(1) process as defined by the following equation:

(5) $$X\left(i\right)=b\cdot X\left(i-1\right)+\varepsilon ,\;\mathrm{with}\;0<b<1,\;\varepsilon ∼N\left(0,\sigma_{\varepsilon }\right).$$

The second time series Y(j) is subsequently constructed based on X(i), while simultaneously inserting an a priori known lag structure τ(j) [38]:

(6) $$Y\left(j\right)=a\cdot X\left(j-\tau \left(j\right)\right)+\eta ,\;\mathrm{with}\;\eta ∼N\left(0,\sigma_{\eta }\right).$$

Following Meng et al. [6], Sornette and Zhou [38], the strength of the lead-lag relationship is thereby mainly determined through the parameters *a* and $f=\frac{\sigma_{\eta }}{\sigma_{\varepsilon }}$, where *a* changes the strength of the transmission from *X* to *Y* and *f* the extent of noise that overlays the relationship. Thus, a higher *a* increases the dependence, while higher values of *f* achieve the opposite.

This manuscript follows Sornette and Zhou [38], Stübinger [43] in setting parameters a=0.8 and b=0.7. While Meng et al. [6], Sornette and Zhou [38] set f=0.2, Stübinger [43] choose f=1. This manuscript chooses a comprise of f=0.5 for the example application. Furthermore, the time-varying lag structure presented in Equation (7) is inserted as in Sornette and Zhou [38] with the extension of one additional period, such that there is no contemporaneous movement at the end and the relaxation of the endpoint constraint becomes relevant:

(7) $$\begin{array}{c} \begin{array}{c} Y\left(j\right)=\left\{\begin{array}{cc} 0.8\cdot X(j)+\eta & 1\leq j\leq 50\\ 0.8\cdot X(j-10)+\eta & 51\leq j\leq 100\\ 0.8\cdot X(j-5)+\eta & 101\leq j\leq 150\\ 0.8\cdot X(j+5)+\eta & 151\leq j\leq 200\\ 0.8\cdot X(j)+\eta & 201\leq j\leq 250\\ 0.8\cdot X(j-5)+\eta & 251\leq j\leq 300 \end{array}\right. \end{array} \end{array}$$

**Figure 5:**
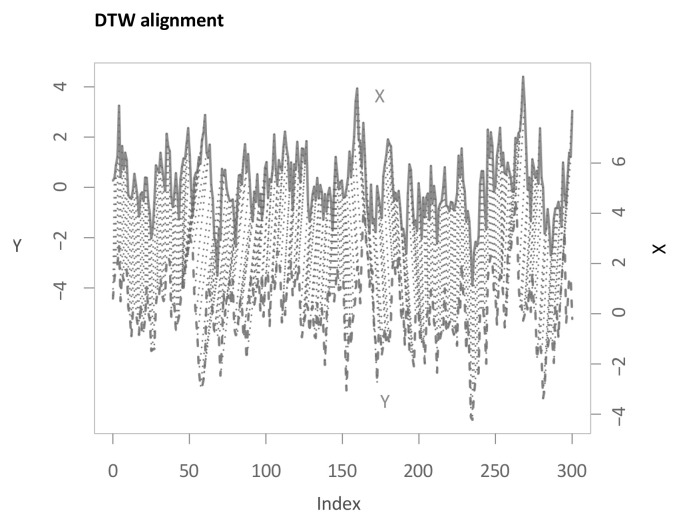
Alignment of the realizations of the two time series *X* and *Y* (see Equation (7)) through the first main step.

Now, we apply our algorithm outlined in Section 3.1. The parameter of the subsequence length *l* from shapeDTW is chosen to be 3 in this first step. Again, a more detailed analysis for varying parameters is presented in the upcoming sections. Performing the first main step of the main methodology then results in the following multi-dimensional DTW alignment (see Figure 5). For the example applications, the boundary condition is relaxed for 30 time steps—more than necessary—to show the functioning of this relaxation for all methods. As can be seen from the graphic, the algorithm makes use of the relaxed endpoint constraint as the last few elements in *X* are not considered.

**Figure 6:**
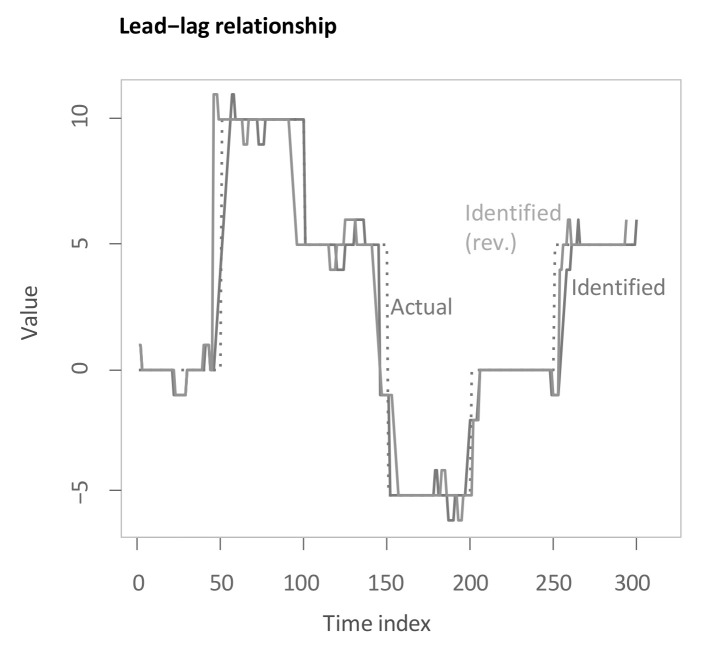
Time-dependent lag structure estimate through main methodology.

Subsequently, computing the second main step of the main methodology yields the following estimate for the lag structure as the solid line “Identified” (see Figure 6). The inserted underlying lag structure is delineated alongside as a dashed line. The estimated lead-lag relationship shows a rather close matching of the underlying lag structure. The algorithm achieves both accurate identification of transitions between different lead-lag regimes with some short prolongations and of stable regimes with some small deviations. To legitimise the drawback of the main methodology that it is not symmetric after conversion to the inputted time scale, the lag structure is additionally presented with reversed insertion for performing the second main step (“Identified (rev.)”). As visible, the output is slightly changed for the transition periods, yet, overall, the difference is very small. This mitigates potential criticism of the asymmetry in inserting both time series. Moreover, in the more extensive simulation study in Section 5.2, again reversion of the insertion is considered. It should be noted that we aim to identify historical lead-lag relationships rather than to predict future patterns.

Processing on a contemporary Intel core i7-6700 HQ with a clock speed of 2.6 GHz leads to an approximate run-time of 1 s. Therefore, applying our algorithm to medium length time series is quite feasible.

## 4. Benchmarks

This chapter presents already existing methods for detecting time-varying lead-lag relationships. More precisely, we discuss Thermal Optimal Path (TOP), Symmetric Thermal Optimal Path (TOPS) (Section 4.1), Rolling Cross-Correlation (RCC) (Section 4.2), and Dynamic Time Warping (DTW), Derivative Dynamic Time Warping (DDTW) (Section 4.3). To evaluate the value-add of our algorithm presented in Section 3, these approaches will then later be utilised for comparisons with the main methodology.

### 4.1. Thermal Optimal Path (TOP), Symmetric Thermal Optimal Path (TOPS)

While the main methodology presented in Section 3 focuses mainly on increasing the information contained in the local cost matrix, the “Thermal Optimal Path method” (TOP) by [6,38] and its revision named “Symmetric Thermal Optimal Path method” (TOPS) by [6] method aim at extracting the relevant information out of the local cost matrix more robustly (see Section 2).

**Figure 7:**
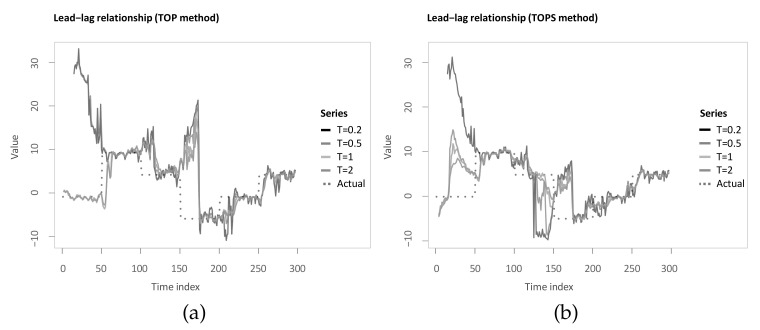
(**a**) Time-dependent lag structure estimate with the Thermal Optimal Path (TOP) method for different temperatures; (**b**) time-dependent lag structure estimate with the Symmetric Thermal Optimal Path (TOPS) method for different temperatures.

Identical to the previous chapter, we apply the described methods to the example in Section 3.3. To be more specific, the TOP and TOPS method are computed for temperatures T=0.2, T=0.5, T=1 and T=2 analogously to Sornette and Zhou [38] (see Figure 7a,b). When comparing the estimated lag structures, it can be seen that, first, both TOP and TOPS overall follow the underlying lead-lag relationship less closely, and the estimated lag structure is considerably more volatile. In particular, for the first 50 time steps, this application shows the value of increasing the temperature to at least 1 for TOP and at least 0.5 for TOPS.

### 4.2. Rolling Cross-Correlation (RCC)

Next to the TOP and TOPS method, the main methodology is benchmarked against three more basic approaches. The first benchmark as in Sornette and Zhou [38] is Rolling Cross-Correlation (RCC). Following Sornette and Zhou [38], the idea is to perform cross-correlation analysis in rolling windows over both series. The rolling windows for both time series are centred at each index and are then shifted by steps of 1. Each time, the cross-correlation function is computed and the lag or lead with the highest correlation is taken as an estimate for the lag structure at the respective index. RCC as a benchmark is computed for different window sizes. In general, longer window sizes lead to more robust estimates as the effect of single noise realisations decreases and shorter window sizes are able to better capture changes in the lag structure. Therefore, a compromise between both competing aims should be selected [38].

**Figure 8:**
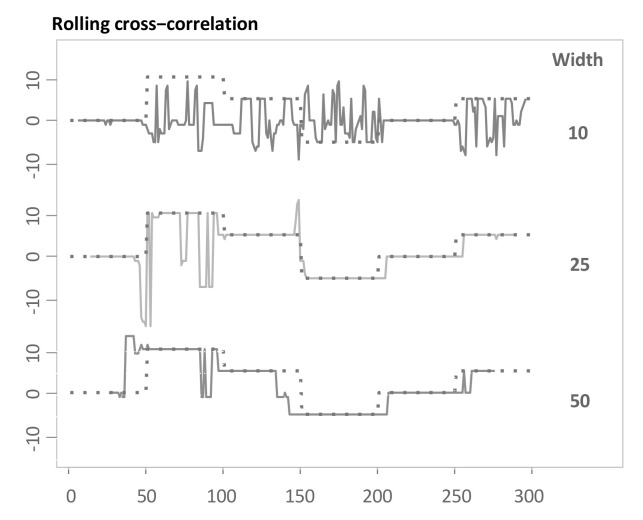
Time-dependent lag structure estimate through Rolling Cross-Correlation (RCC) for different window sizes.

Figure 8 visualizes the outputted lead-lag relationships after applying this method to the example in Section 3.3. To be more specific, we use different window sizes of either 10, 25 and 50. The method works reasonably well for window sizes of 25 and 50. There is a compromise in choosing the optimal window size as also noted by Sornette and Zhou [38]. For short window sizes, the approach is highly prone to the influence of noise and for large window sizes, and the algorithm requires a substantial amount of start-up period and similarly at the end, and is less responsive to regime changes.

### 4.3. Dynamic Time Warping (DTW), Derivative Dynamic Time Warping (DDTW)

As a second and third benchmark function, the lead-lag extraction based on classical DTW and DDTW, respectively. As already mentioned, these algorithms or modified versions have already been applied to the lead-lag relationship by Varfis et al. [41], and Claure et al. [45], Woo et al. [46], respectively. Classical DTW, DDTW and the main methodology all have distinctive steps for calculating the local cost matrix. Then, after the calculation of the local cost matrix, the remaining steps as presented for the main methodology are performed equally upon the local cost matrices calculated by classical DTW and DDTW in order to keep the methods as comparable as possible.

**Figure 9:**
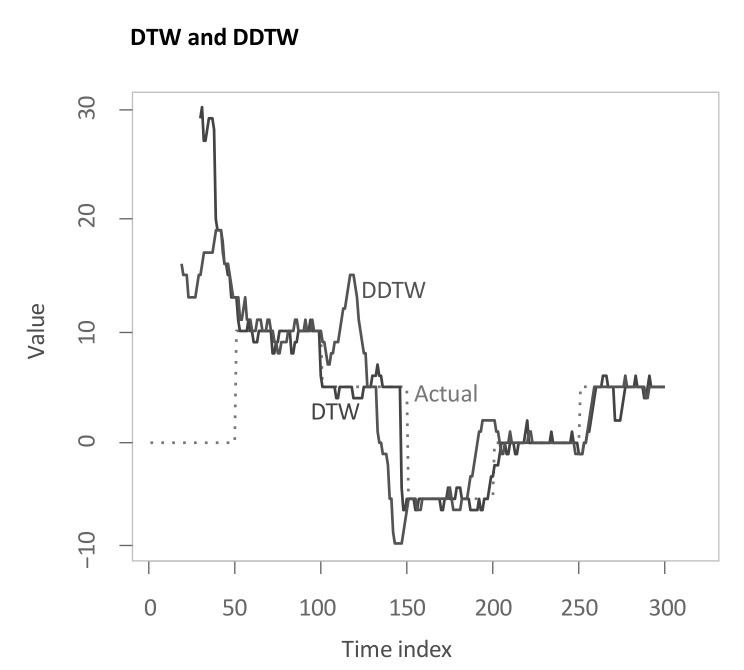
Time-dependent lag structure estimate through Dynamic Time Warping (DTW) and Derivative DTW (DDTW).

Again, the resulting output is shown in Figure 9. Both algorithms struggle to find the correct lag estimate for the first 50 time steps. For the remainder, both produce rather close estimates. However, while DTW is more volatile during stable regimes, DDTW faces some problems in the transitions between regimes.

Thus far, the methodologies are concerned with an example application on a single pair of simulated data to give an overview of the outputs of these methods. The following section aims at drawing a more holistic and robust picture by judging the methods based on an extensive amount of simulated data with different parameter constellations for generating the simulated data.

## 5. Simulation Study

This chapter compares and evaluates the functioning of the main methodology and the alternative methods presented in Section 3 and Section 4, respectively. For this purpose, the assessment criterion is introduced (Section 5.1), and the methods are then evaluated based on it (Section 5.2). Finally, Section 5.3 concludes with further robustness checks, and Section 5.4 presents a real-word application.

### 5.1. Assessment Criterion

**Figure 10:**
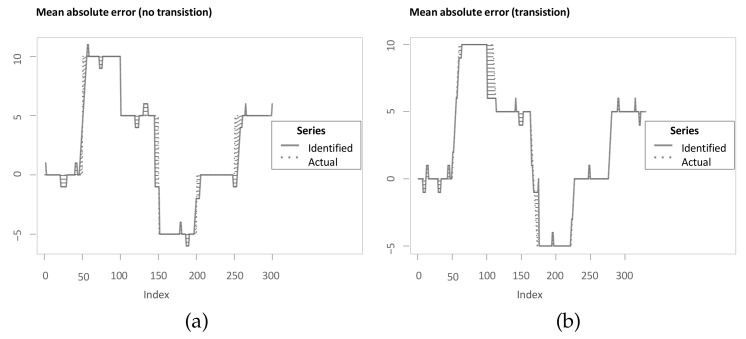
(**a**) Mean absolute error (MAE) without transition between different regimes; (**b**) mean absolute error (MAE) with transition between different regimes.

Since the aim of the methods is to accurately identify the underlying lag structure, the assessment criterion is based on the mean absolute error (MAE) between the identified lag structure and the inserted lag structure. In this manuscript, the term MAE refers to the mean of the absolute differences between identified and underlying lag structure at all indices. An advantage of the MAE is that it can be interpreted straightforwardly in that it measures the average difference in both directions from the “true” underlying lag structure. In addition, Zhao and Itti [4] use the MAE for comparing optimal warping paths to simulated scaled and stretched equivalents, however without considering noise masking the relationship. For the assessment criterion in this manuscript, two modifications are integrated to improve it. Firstly, the measure is to a large degree driven by the periods of transition between different lags or leads (see Figure 10b). While this might be plausible, there is a drawback in the way the simulated data are generated. Referring back to the example application and the inserted lag structure presented in Equation (7), it can be seen that jumps in the inserted lag structure contradict the idea of a continuous underlying lead-lag relationship. For instance, the first jump from time step j=50 to j=51 leads to a situation where Y(50) is already dependent on X(50), with previous time steps being contemporaneous as well and then Y(51) becomes dependent on X(41) and thus the dependence jumps back in time. Interestingly, Sornette and Zhou [38] justify the optimal mapping in the spirit of warping paths in that the monotonous and step size condition are essential to ensure no volatile lag structure and no jumps back in time once two indices are matched. However, the simulation study presented in Sornette and Zhou [38] suffers from this drawback. Therefore, the simulation is adapted, such that a continuous transition between jumps in the lag structure is inserted. For instance, between time steps j=50 and j=51, an additional nine time steps are inserted with lag values of [1,2,⋯,9] to create a smooth transition. Thus, the underlying lag structure is either stable with a slope of 0 or it is increasing or decreasing with a slope of +1 or −1, respectively (see Figure 10a). Again, for two of the transitions, the estimate is not corresponding exactly. However, the MAE is less driven by periods of transitions. Additionally, a perfect alignment would be possible, which has another benefit. Namely, an MAE of 0 is possible for all methods and optimal and therefore the values can be interpreted with respect to that.

**Figure 11:**
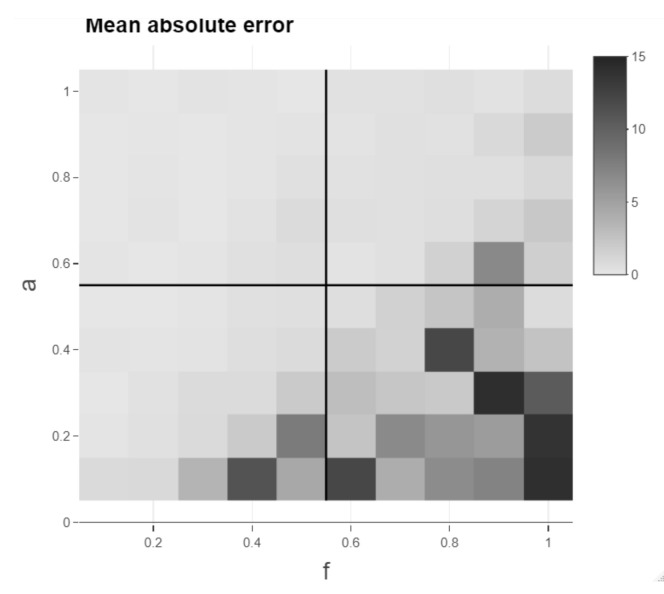
Heatmap of mean absolute error (MAE) for different combinations of *a* and *f*.

Moreover, basing the assessment of the methods on a single time series pair realisation makes it more prone to data snooping and the specific realisation and, additionally, the methods are then only performed on a single parameter combination of *a* and *f*. In order to mitigate these two drawbacks, the parameters *a* and *f* are varied between 0.1 and 1 in steps of 0.1, respectively. Meng et al. [6] also vary both *a* and *f* in their analysis of their novel significance test. For each combination of the parameters, a pair of time series is simulated with smooth transitions as mentioned before and a lag structure with five stable regimes of 50 time steps each, where each of the five lead-lag values is randomly drawn in the interval between −10 and 10 to create an underlying time-varying lead-lag relationship. Random draws of lead-lag values are also done in Meng et al. [6]. The relaxation of the endpoint constraint is applied for all presented methods (except RCC) equivalently with a relaxation of 10 possible time steps for one of the series at the beginning and one at the end of the alignment. The number 10 is chosen to be high enough to allow the mean of MAE over 100 different realisations of time series pairs (MoMAE) to be 0 for perfect alignments and low enough such that the extent of the computations is feasible on a single computer. In case, the then computed lead-lag estimate starts later or ends earlier than the underlying lag structure, it is padded with NAs in the code, and these NAs are omitted in both the estimated and inserted lag structure for the calculation of the MAE. The output of the calculations can be visualised with the help of a heatmap (see Figure 11). The heatmap is then divided into four quadrants (low *a* and low *f*; high *a* and low *f*; low *a* and high *f*; high *a* and high *f*), where high refers to values from ]0.5;1] and low to ]0;0.5]. For each quadrant, the mean over all calculated MAE is computed as well as the mean over all quadrants. These key figures then serve as assessment criteria and are referred to as a mean of MAE (MoMAE). Each assessment criterion contains various different lag structures, various combinations of *a* and *f* and thus averages over multiple realisations to reduce the dependence on single realisations. In order for the comparison to be consistent, the 100 different realisations of time series pairs are the same for all methods. Furthermore, by dividing the MAE into four quadrants, an analysis and comparison for different strengths of the lead-lag relationship can additionally be performed. As for the MAE, the MoMAE is equal to zero at the best and generally the lower the MoMAE, the better. In further research, potentially, one could think of constructing confidence intervals based on the MAE or MoMAE.

### 5.2. Assessment of Methods

In this section, the main methodology and alternative methods are assessed based on the criterion presented in the previous section. Thereby, the main methodology is calculated once without concatenating the subsequence with the first differences (MM) and once with concatenation (MM(w.diff)). While the alternative methods DTW and DDTW do not have a parameter attached to them, the other five methods (MM, MM(w.diff), TOP, TOPS, RCC) each have one parameter. To be able to compare the different methodologies, the assessment criterion is calculated for a comprehensive set of different values of the respective parameters. The parameters chosen for both main methodologies are subsequence lengths *l* from 1 to 31 in steps of 2 for MM and 3 to 31 in steps of 2 for MM(w.diff), as for 1, no first difference can be calculated with only one value. For both TOP and TOPS, temperatures *T* from 0.5 to 2.3 by steps of 0.3 are used as comparably done by Meng et al. [6]. Finally, for RCC, values from 10 to 60 in steps of 5 are taken for the size of the rolling windows. As mentioned above, all seven methods are performed on the same realisation to allow for comparability.

**Figure 12:**
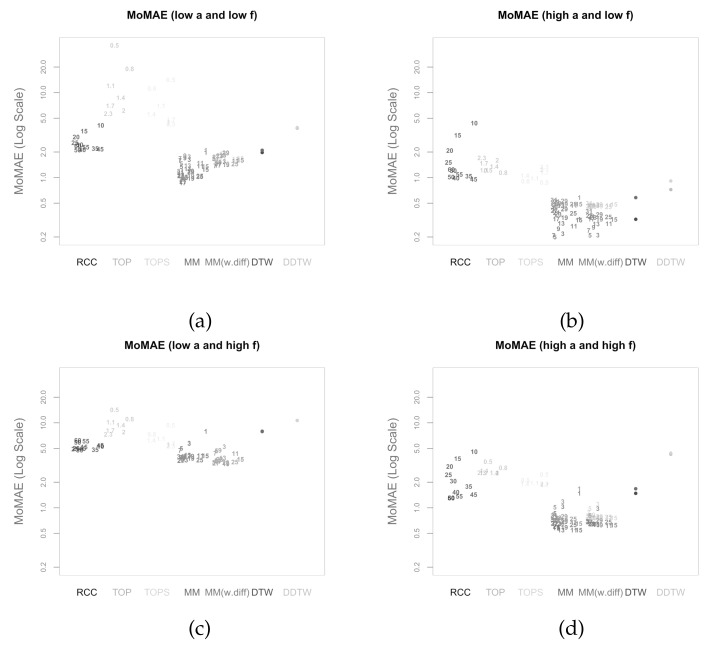
Mean of MAE (MoMAE) for (**a**) low *a* and low *f*; (**b**) high *a* and low *f*; (**c**) low *a* and high *f*; (**d**) high *a* and high *f*. Numbers characterize the value of the corresponding parameter.

Figure 12a–d present the respective MoMAE for all methodologies for different parameter settings (*a* and *f*) in order to analyze several parameter constellations—this procedure depicts possible impacts of the hyperparameters [64]. Since there are seven methods, five of them with several parameter constellations and four different quadrants, the figures are slightly complex at first sight and need some introduction. As mentioned, each of the four quadrants over the MAE is portrayed as a separate figure. In each figure, the different methods are displayed on the horizontal axis each with its own distinct colour. On the *y*-axis, the respective MoMAE as assessment criterion is shown. Importantly, the *y*-axis is displayed in log scale in order to better distinguish the various values. For each parameter value of the methods, a single entry is plotted as text containing the respective parameter value. The jittering along the horizontal dimension is done to increase readability. For the four methods on the right, the entries are duplicated with the respective results portrayed in a lighter colour for the reversed insertion of the time series. Therefore, it is important for the latter four methods to only compare lighter colours to lighter colours and darker ones to darker ones. The lighter colours generally portray values higher than the darker correspondences as within the simulated data a clear directed dependence of one time series to the other is inserted. An elegant solution to overcome the symmetry problem is possible in case the two time series are of equal length. Then, one can average the lead-lag estimate over both estimate series at every index and one would obtain an MAE and thus MoMAE lying in the worst case in the middle of the respective lighter and darker colours. Most likely, however, the estimate would be better or rather lower than the mean between the MoMAE as in instances where one estimate is overestimating and the other underestimating, the MAE of the averaged estimates is lower than the average of the MAEs. This can be considered in further research as an extension to the main methodology that is possible for equal-length time series.

The following paragraph is dedicated to describing the results that can be derived from the four subfigures in Figure 12. In general, the worst MoMAE over all methods are found for low values of *a* in combination with high values of *f* with the exception of TOP and TOPS that struggle for some parameter constellations even stronger with low *a* and low *f*. The best results are clearly found for high *a* and low *f*. This is completely in line with what is expected, as strong relationships with little noise should be easier to identify than weak ones overlaid with more noise. Importantly, the analysis shows that the main methodology both with and without first difference outperforms other methodologies for practically every parameter constellation with two exceptions, where some parameter values achieve second and third best. Namely, in case of low *a* and high *f* for low parameter values, the RCC and TOPS method obtain slightly better results and for high *a* and low *f* classical DTW surpasses MM and MM (w.diff) with higher parameter values. Still, the best MoMAE is always found for parameter values of one of both main methodologies.

For the strongest relationships (high *a*, low *f*), the values of the MoMAE found for both main methodologies are between 0.2 and 0.6, suggesting that the identified lag structure is on average continuously very close to the underlying one. Similar for high *a* combined with high *f*, the identified lag structures deviate from the underlying one on average by 0.5 to 1 for both main methodologies. In case of low *a* and low *f*, the MoMAEs increase for the main methodologies to values between slightly below 1 up to 2, still allowing one to interpret estimated lag structures quite well. For the instances with low *a* and high *f*, then the values of MoMAE become relatively high, such that lag estimates should be interpreted cautiously, as the identified lead-lag relationship deviates on average by values from 3 to slightly above 5. Obviously, the same should be considered for the alternative methods, as some of these surpass values of 3 already for stronger relationships. It is interesting to note that MM with the parameter of the subsequence length equal to 1 is identical to classical DTW, which is why the MoMAE for MM with parameter set to 1 achieves the exact same results as DTW. A very positive finding is that the MoMAE for both main methodologies are very similar for all parameter constellations ≥3, indicating that the method is superior on average without the need to perform an excessive hyperparameter tuning to surpass other methods. If one compares the two different main methodologies, one can find that the results lie very close to each other, where MM(w.diff) performs slightly better for low *a* and high *f* and slightly worse for low *a* and low *f*. Therefore, in the pursuit of parsimony, the results so far do not suggest additionally including first differences.

The assessment also underlines the findings in Meng et al. [6], Sornette and Zhou [38] that, for the TOP and TOPS method, the parameter *T* should be chosen close to two for most constellations. Moreover, as for MM and for the TOP and TOPS method, higher parameter values lead to an increasing robustness against noise at the cost of small precision losses. Therefore, these higher parameter values obtain better or rather lower MoMAE for weaker underlying lead-lag relationships, while for strong relationships they come second after smaller parameter values. Moreover, TOP and TOPS perform comparatively well for low *a* and high *f*, which underlines one idea behind TOP and TOPS, where the introduction of temperature is explained through mitigating the interfering of noise [6,38]. However, as for these weak relationships, the MoMAE lies around 5, the question arises how useful the estimated lag structures are. Moreover, the figures show that the TOPS method generally outperforms its predecessor, the TOP method, which might be due to more robust estimates that consider the whole local cost matrix, thus affirming Meng et al. [6].

In the above presented assessment, some assumptions are drawn that might be unrealistic. One of these is the length of the stable regimes of the lag structure. Namely, the underlying lag structure is stable for 50 time steps, then smoothly changes to a different lag regime and is stable again for 50 time steps. This assumption is potentially problematic in the application for real world data. In particular, in the case of monthly or quarterly data, this would assume a stable lead-lag relationship for a substantial time duration. Moreover, the transmission of time series *X* on *Y* before noise is constant throughout the two time series. However, in real world data, the strength of the transmission can be time-varying as well. Additionally, one important assumption is that the noise added to the second time series *Y* is made up of white noise, which slightly favours methods that internally average over the noise such as the main methodology or RCC. In order to show that the results presented within this section are robust towards changes in these assumptions, the next section presents three robustness checks.

### 5.3. Robustness Checks

In pursuit of consistency and clarity, the following robustness checks are again based on the MoMAE as assessment criterion with the difference that this time the MoMAE is not distinguished for each quadrant but is computed as mean over all calculated MAE.

**Figure 13:**
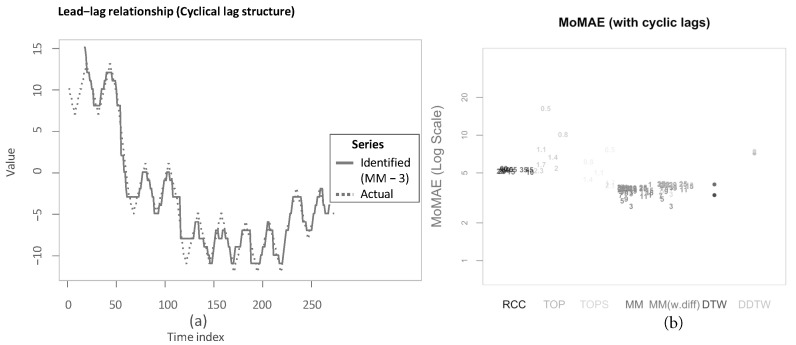
Robustness check towards cyclical lag structure. (**a**) lead-lag relationship with cyclical lags; (**b**) MoMAE with underlying cyclic lag structure.

As a first robustness check, the different methods are tested upon underlying lag structures that have an added cyclical component. This therefore ensures that the lag structure is not fixed for the prior stable regimes but fluctuates around a given value before transitioning to the next regime. Figure 13a shows one example application of MM with subsequence length 3. As is visible, the identified lead-lag relationship follows the actual one very closely. Then, Figure 13b shows the results of the simulation. As can be seen, both MM and MM(w.diff) are performing comparatively well for all parameter constellations. MM and MM(w.diff) with subsequence length set equal to 3 perform the best overall. This is owing to the quickly changing lag structure, as small parameter values for the main methodologies are better to follow frequent changes in the lag structures accurately and swiftly. However, subsequence lengths of 3 and 5 still dominate subsequence lengths of 1 (which equals classical DTW), which again underlines the importance of neighbourhood information within the local cost matrix. Still, classical DTW outperforms higher values of the subsequence lengths. It must be noted that the values of the MoMAE are on average rather high. In the simulations in the section before, MM achieves over all four quadrants and over all subsequence lengths an average of 1.67 and 1.77 with reversed insertion. MM(w.diff) achieves 1.62 and 1.72, respectively. The values for this robustness test are notably larger for both main methodologies, suggesting that likely not all cyclicalities could be fully reproduced.

**Figure 14:**
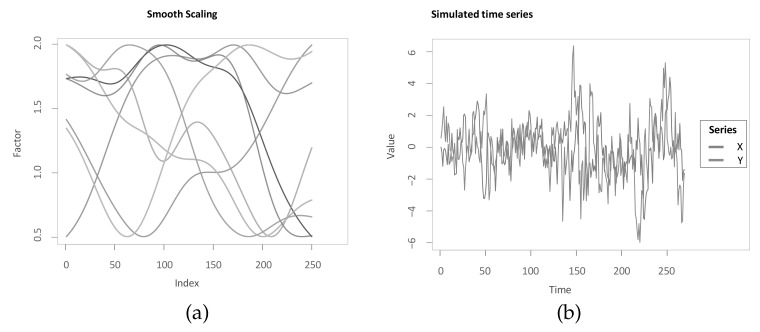
Robustness check towards smooth scaling. (**a**) smooth scaling series; (**b**) simulated time series with smooth scaling.

The second robustness check tests the functioning of the methodologies in case the transmission of one time series upon the other changes over time. Within the simulation study, the effect of a change in *X* upon the value of *Y* is given and with added white noise, the Z-normalization is then well able to achieve that corresponding phases share very similar values. However, this might be an unrealistic assumption in the real world, as the transmission of the effect may change over time. Therefore, this second robustness check follows in a similar manner an idea of Zhao and Itti [4] in scaling the transmission from *X* to *Y*. Therefore, the parameter *a* is scaled over time and thus indirectly also the time series. Zhao and Itti [4] argue that the series with which is scaled needs to be smooth, since otherwise the scaling potentially completely changes the nature of the time series. While Zhao and Itti [4] uses random normally distributed numbers and applies the sinus function on the cumulative sum to recursively achieve smoothness, for this manuscript, the cumulative sum over random draws from an uniform distribution between −1 and 1 is smoothed using a Gaussian kernel smoother. Finally, following Zhao and Itti [4], the smooth series are then scaled such that they span a given interval. While Zhao and Itti [4] take the interval from 0.5 and 1, this manuscript takes the interval from 0.5 and 2, such that the scaling works in both directions. For only the interval between 0.5 and 1, the effect of this addition would be already partly mitigated through the Z-normalization. Figure 14a shows, as similarly done in Zhao and Itti [4], eight different realisations of the random smooth scaling series used in this manuscript. In Figure 14b, a simulated time series pair is shown, where the transmission of *X* upon *Y* is scaled as $Y\left(j\right)=a\cdot S_{Scale}\left(j\right)\cdot X\left(j-\tau \left(j\right)\right)+\eta ,\;\mathrm{with}\;\eta ∼N\left(0,\sigma_{\eta }\right)$, where $S_{Scale}$ is one realisation of a smooth scale series. This transformation of the second time series might potentially destroy the stationarity of *Y*, as it might change the co-variance of given lags over time. However, performing the Phillips–Perron test and the KPSS test on the time series at each combination of *a* and *f* leads to a rejection of the null hypothesis of the Phillips–Perron test on the 1% significance level for 100 out of 100 time series, and the KPSS test can not reject its null hypothesis at the 10% level for 73 out of 100 time series. This overall suggests that the scaling does not interfere too strongly with the stationarity. In any case, the following results show that the main methodology still works.

**Figure 15:**
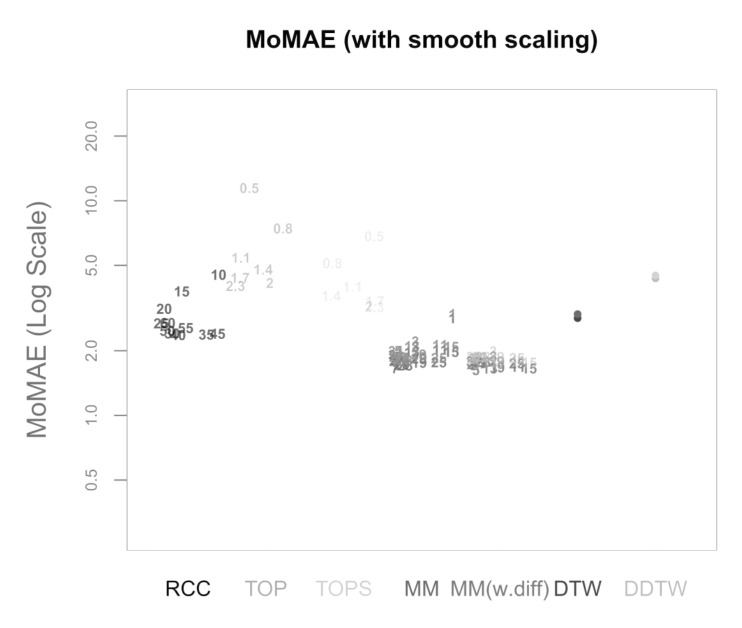
MoMAE with underlying smooth scaling.

Assessing this based on the MoMAE yields the following Figure 15. Again, both main methodologies outperform the other methodologies. As visible, MM(w.diff) is marginally better than MM, which might be explained by the fact the inclusion of smooth scaling leads to a situation where similar progressions do not share very close values on average, but similar phases exhibit more different values. Therefore, adding the information contained within a value-independent measure of shape such as the first differences is marginally improving the results. For this robustness check, the absolute values of MoMAE for both main methodologies are almost in line with the ones achieved in Section 5.2.

**Figure 16:**
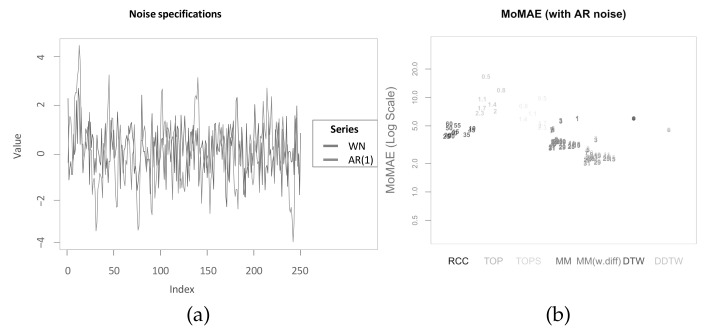
Robustness check towards different noise specification. (**a**) Different noise specifications; (**b**) MoMAE with underlying AR noise.

The third and final robustness check concerns the assumption that the noise added upon the second time series is white noise, and there is thus no autocorrelation within the noise. However, for real world time series, one could assume the additional noise to be autocorrelated as well. For instance, *Y* might be dependent on *X*, but also upon an unknown *Z* that equally is a first-order autoregressive process. As the lead-lag relationship between *X* and *Y* is studied here, the contribution of *Z* is referred to as noise. Therefore, within this robustness check next to the direct transmission from *X* to *Y*, a random first-order AR process is added with the same model specifications as for *X*. Thus, *Y* is a weighted sum over two independent first-order AR processes that share the same model specification. Subsequently, the lead-lag relationship of *Y* with *X* shall be identified in the presence of the added “AR noise”. The difference between the prior white noise (WN) and the AR(1) process is pictured in Figure 16a. Clearly, the difference in persistence of the fluctuations can be seen. Therefore, one can expect *Y* as the weighted sum to also show persistence for similar phases in the deviations from the absolute levels of *X* after Z-normalization. Computing the assessment criterion upon this specification leads to the following output (see Figure 16b). This time MM(w.diff) is the clear winner, and MM performs comparatively well. However, DDTW, TOPS and RCC outperform MM with small subsequence lengths. The results again suggest that, in case of persistence in the difference of values after Z-normalization, features describing shape information independent of the value level, such as derivatives in DDTW, correlation in RCC and first differences in MM(w.diff) perform comparatively well. This also is the first instance where DDTW outperforms classical DTW. Considering the absolute levels of the MoMAE, this robustness check still suggests that the changed noise specification makes it harder for both main methodologies to identify the “true” underlying lag structure.

Overall, the robustness checks underline the findings of Section 5.2. Both main methodologies are outperforming the alternative methods for the majority of parameter values, and the lowest MoMAE is still always achieved by one parameter setting of MM or MM(w.diff). Again, the MoMAE values over all parameter constellations of the main methodologies are bundled very close. The robustness checks also show that, for all three checks, the levels of MoMAE for both main methodologies rose, indicating that the robustness checks increased the difficulty of accurately and swiftly identifying the actual time-varying lead-lag relationships. Generally, for identifying frequent and short-lasting lead-lag relationships, small values of the main methodology are preferable. Again, small does not refer to values of 1, which would correspond to classical DTW, as the inclusion of neighbourhood information proves valuable basically throughout the simulations. However, for more complex noise patterns, as with "AR noise", higher values of the subsequence length are superior due to increased robustness. The inclusion of first differences becomes valuable in case similar phases persistently deviate in their levels after Z-normalization, where then value-independent shape characteristics become more important. This latter finding is in line with further research on this topic [4,11].

### 5.4. Real-World Application

The purpose of this subsection is to show the performance of the algorithm on real-world data, namely the German and US business cycles, as well as to motivate further research upon business cycle data based on this methodology or DTW approaches in general.

The data set stems from the “OECD Main Economic Indicators database” [65] and contains besides various leading indicators also the original quarterly series on business cycles of the OECD members. The business cycles are thereby measured as the growth rate of real GDP in percent from the corresponding quarter in the previous year as similarly done in Duran and Ferreira-Lopes [66] and are seasonally adjusted [65]. For this manuscript, the quarterly business cycle data are used starting in 1991 up until the second quarter of 2021.

In what follows, the lead-lag relationship between the US and German business cycle is computed. As detailed in the chapters before, the main methodology has especially one parameter, namely the subsequence length *l*. As already shown, the results of different parameters are very close, yet low values are still better able to capture frequent changing lag structures and since, due to quarterly data, the series are observed at a low frequency, this is an important criterion. Therefore, subsequence length 5 is selected, being the second smallest value for shapeDTW with neighbourhood information. Moreover, as Belke et al. [67] mention, business cycles, even strongly synchronised ones, regularly show different amplitudes. Therefore, the option of additionally including first differences is chosen, as the robustness checks show that MM(w.diff) outperforms MM in case of temporally persistent differences in the values.

**Figure 17:**
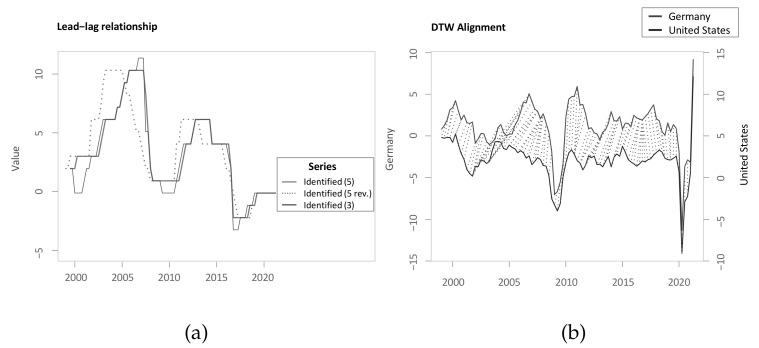
German and US business cycle. (**a**) lead-lag relationship; (**b**) alignment.

Thereby, in order to mitigate the symmetry issue, the lead-lag relationship is plotted as is (“Identified (5)”) and with reversed insertion (“Identified (5 rev.)”) (see Figure 17a). Additionally, the lead-lag relationship using subsequence length of 3 (“Identified (3)”) is also included to show whether the results are robust to slight changes in the parameter. Thereby, positive values of the lead-lag relationship describe the situation, where the US business cycle is estimated to lead the German one and negative values indicate the opposite. Keeping in mind the results of the assessment criterion MoMAE within the simulation study, the estimated values for non-strong relationships showed notable average deviations from the true underlying relationship. Therefore, the concrete values are to be interpreted keeping this in mind and further research should focus on introducing measures for confidence intervals. Figure 17b shows the calculated alignment between the two inputted time series, underlining the alignment quality of the method.

As can be seen from the left figure, the estimated lead-lag relationship (“Identified (5)”) starts with the US increasing its lead over Germany over the whole time period from 1999 until right before the world financial crisis. Then, with the world financial crisis, the lag estimate reduces to a value of 1, indicating that the US business cycle leads the German one by one quarter for around two to three years. After the great recession, the US business cycle picked up to lead the German cycle once again with higher values; before around 2016, the lead changes, and Germany slightly leads the US business cycle. This matches right about the time when US trade in percent of GDP reduces as is visible in [68]. However, it is not the aim of this application to analyse any underlying factors and their connection to the estimated lead-lag relationship. Therefore, the question on why there is an estimated change in the lead between the business cycles is a topic for future research. Finally, starting in the end of 2019 and including the subsequent COVID-19 crisis, both cycles are estimated to be contemporaneous. This generally is an interesting, yet non-surprising observation that the lead-lag estimate changes to contemporaneous or nearly contemporaneous for periods of economic crisis. The reversed lead-lag relationship (“Identified (5 rev.)”) shows a similar picture, yet the estimates are partly shifted in time especially for the period before the financial crisis. Therefore, the estimates should rather be considered over some duration and not independently for each time point. The lead-lag structure estimated with smaller subsequence length (“Identified (3)”) shows a similar picture, with the difference that the recession in the early 2000 is estimated contemporaneous, and the US lead by 1 quarter to the start of the global financial crisis reduces to contemporaneous quickly. These two changes might result from the increased adaptability to short changes in the lag structure also seen in the simulation study.

## 6. Discussion and Conclusions

As elaborated in the preceding chapters, the inclusion of shapeDTW as presented in Zhao and Itti [4] towards the application on lead-lag relationships offers a large potential for improving the accuracy of the lead-lag estimates. The large-scale simulation study demonstrates that this method is systematically able to outperform currently used methods such as TOP and TOPS. The results are thereby robust towards different specifications of the noise, scaling of the relationship and frequent changing lag structures.

As part of this conclusion, especially six directions for further research are highlighted. Firstly, as mentioned by Zhao and Itti [4], the dimensions could be weighted differently. This possibility has not been considered in this manuscript. Secondly, as noted a few times, the simulation study showed that there is a notable average deviation from the inserted lag structure for non-strong relationships. Therefore, the introduction of a reliable confidence interval around the lead-lag estimate would be very informative. Thirdly, the main methodology needs to be evaluated upon more diverse (real-world) data and metrics. In particular, for a given thematic application on real world time series, it might prove very useful to first fit ARIMA models to the original data, then simulate new data using the estimated models, such that the results of the simulations are as close to the actual data as possible. Within the simulation study of this manuscript, the time series are based on first-order AR processes with a coefficient of 0.7, such that results for the main methodology might deviate for different ARIMA processes, especially with higher persistence. This would allow for drawing more meaningful conclusions in further applications. Furthermore, we could also vary the choice of some inputs, e.g., use Root Mean Squared Error instead of Mean Absolute Error, to analyze the influence of inputs of our framework. Fourthly, a potentially very valuable contribution to the literature might be found for combining shapeDTW by Zhao and Itti [4] for creating the local cost matrix with the extraction of the lead-lag relationship through the TOP or TOPS method detailed in Meng et al. [6], Sornette and Zhou [38], as the approaches are generally complimentary. To the best of our knowledge, there has not been published research on combining these two research strands. Only Meng et al. [6] mention in one sentence that the TOP method might work with other distances as long these are “sufficiently local” [6] (p. 961). It would be interesting to see whether a potential combination leads to a further improvement. In the future, the methods can also join the research strand that applies DTW upon forecasting time series in case of stable or other predictable time-varying lead-lag relationships. Fifthly, another promising research direction is to decompose each time series into the time-frequency domain and then estimate the lead-lag (time delay) between the harmonics of the two time series [69,70,71]. Therefore, we might obtain higher density of information and consequently a better alignment between both time series. Finally, we could increase the performance in the context of big data. Assent et al. [72] propose a novel filter-and-refine DTW algorithm called Anticipatory DTW. The implemented algorithm aims to efficiently find similar time series by filtering the database and computing the DTW in the refinement step.

## Data Availability

The data presented in this study are openly available in the OECD Main Economic Indicators-Complete database at https://doi.org/10.1787/data-00052-en [65] (accessed on 18 June 2022).
